# The E3 ubiquitin ligase MuRF2 attenuates LPS‐induced macrophage activation by inhibiting production of inflammatory cytokines and migration

**DOI:** 10.1002/2211-5463.12367

**Published:** 2018-01-08

**Authors:** Hongjun Bian, Shanshan Gao, Di Zhang, Qi Zhao, Feifei Li, Xiao Li, Shuohuan Sun, Shouyang Song, Tao Li, Qiang Zhu, Wanhua Ren, Chengyong Qin, Jianni Qi

**Affiliations:** ^1^ Shandong Provincial Hospital Affiliated to Shandong University Jinan China; ^2^ Shandong Provincial Engineering and Technological Research Center for Liver Diseases Prevention and Control Jinan China

**Keywords:** hepatitis, lipopolysaccharide, macrophage, muscle RING‐finger 2, nuclear factor‐κB

## Abstract

Muscle RING‐finger (MuRF) proteins are E3 ubiquitin ligases that are expressed in striated muscle. MuRF2 is an important member of this family, but whether it is expressed in tissues other than striated muscle has not been thoroughly elucidated to date. In this study, we determined that MuRF2 is also expressed in other vital organs, including liver, lung, brain, spleen and kidney. Moreover, we show that the level of MuRF2 expression is significantly decreased in hepatic mononuclear cells of mice with lipopolysaccharide (LPS)/d‐galactosamine‐induced hepatitis and negatively correlated with the serum levels of alanine aminotransferase and aspartate aminotransferase in these mice. Furthermore, the expression of MuRF2 was down‐regulated in RAW264.7 cells activated with LPS but not in cells treated with polyinosinic‐polycytidylic acid (Poly(I:C)) or with lipidosome plus Poly(I:C). We also found that MuRF2 was able to translocate from the cytoplasm to the nucleus in RAW264.7 cells activated with LPS but not in cells treated with Poly(I:C). In addition, we demonstrated that interleukin 6 and tumour necrosis factor α production and macrophage migration were inhibited after MuRF2 was overexpressed in RAW264.7 cells. We further verified that nuclear factor‐κB p65 subunit level was greatly reduced in RAW264.7 macrophage nuclei by gain of function. Taken together, these findings indicate that MuRF2 may rescue LPS‐induced macrophage activation by suppressing the production of proinflammatory cytokines and cell migration. We also identify a novel function of MuRF2 in non‐muscle tissues and cells.

**Abbreviations**
ALT: alanine aminotransferase
AST: aspartate aminotransferase
d‐GalN: d‐galactosamine
GAPDH: glyceraldehyde‐3‐phosphate dehydrogenase
HMC: hepatic mononuclear cell
IFN‐β: interferon β
IL: interleukin
IκB: inhibitor of NF‐κB
LPS: lipopolysaccharide
MG132: *N*‐carbobenzoxyl‐l‐leucinyl‐l‐norleucinal
MuRF: muscle RING‐finger
NF‐κB: nuclear factor‐κB
PDLIM2: PDZ and LIM domain 2
Poly(I:C): polyinosinic‐polycytidylic acid
PPAR‐γ: peroxisome proliferator‐activated receptor γ
RIG‐I: retinoic acid‐inducible gene I
SOCS‐1: suppressor of cytokine signaling 1
SRF: serum response factor
TLR: toll‐like receptor
TNF‐α: tumor necrosis factor α

The muscle RING finger (MuRF) family is a subfamily of TRIM/RBCC proteins that is characterized by its tripartite motif, including a RING finger, B‐box and coiled‐coil region 1. The MuRF family consists of three members (MuRF1–3) that have been identified as E3 ubiquitin ligases participating in a wide range of biological processes via their RING finger 2, 3. To date, as the name suggests, it was thought that the MuRFs were expressed specifically in striated muscle, including skeletal and cardiac muscle 3. MuRF1 has been well investigated as a crucial regulator in skeletal muscle atrophy by targeting associated proteins, primarily including titin, as reviewed by Rom and Reznick 4, 5. In addition, MuRF1 but not MuRF2 plays an important role in mediating the induction of dilated cardiac hypertrophy in mice after increased pressure, likely by MuRF1's direct interactions with serum response factor (SRF) 6. MuRF3 is thought to be associated with the stability of microtubules, which is important for muscle differentiation 7. MuRF2 is the least characterized member of the MuRF family, containing at least four isoforms because of differential splicing at the C termini of *MURF* genes in humans 8. While three of the isoforms, including p60A, p60B and p50A, are expressed in skeletal muscle, the smallest isoform, a 27 kDa peptide, was specifically expressed in cardiac muscle 8, 9. The smallest isoform lacks a coiled‐coil domain and therefore cannot homo‐ or hetero‐oligomerize. In addition to participating in sarcomere assembly by mediating the transient association of microtubules with myosin and titin, MuRF2 plays potential roles in signal transduction and transcription regulation through stress‐induced translocation to nuclei in cardiac myocytes 8, 9. For example, MuRF2 nuclear translocation in response to mechanical inactivity decreases the nuclear transcription factor SRF and inhibits transcription 10.

To date, no studies have investigated whether MuRFs are also expressed in tissues other than striated muscle and, if so, what roles they may play in the specific tissue. In regard to MuRF2 in particular, as a regulator of signal transduction and transcription in cardiac myocytes, we wondered whether it affects the signal transduction process in other tissues. All of these questions need to be further studied, which may modify perception of the MuRF family and open up new prospects for the treatment of human diseases.

Lipopolysaccharide (LPS)/d‐galactosamine (d‐GalN)‐induced hepatitis in mice is a widely used experimental animal model for research into acute hepatitis pathogenesis and therapies 11, 12. In this model, the activation of macrophages induced by LPS, whose toxic effects can be enhanced by d‐GalN, plays a crucial role in the pathogenesis of hepatitis. Toll‐like receptor (TLR) 4, a transmembrane protein expressed on macrophages, interacts with LPS, activates the intracellular signaling transduction pathway and leads to the phosphorylation and nuclear translocation of nuclear factor‐κB (NF‐κB). NF‐κB initiates the production of proinflammatory cytokines, such as tumor necrosis factor α (TNF‐α), interleukin (IL) 6 and IL‐1β 13, 14, 15, 16. NF‐κB inhibitors have been widely used in anti‐inflammatory therapies, including those to treat hepatitis 17. In addition, E3 ubiquitin ligases, such as PDZ and LIM domain 2 (PDLIM2), suppressor of cytokine signaling 1 (SOCS‐1) and peroxisome proliferator‐activated receptor γ (PPAR‐γ), have been demonstrated to inhibit the activity of NF‐κB and are thought to act as potential targets for anti‐inflammatory therapies 18, 19, 20. However, whether MuRF2 affects NF‐κB activation is still unknown.

In this study, we explored the expression and function of MuRF2 in tissues other than striated muscle. Our research observed that MuRF2 is also expressed in the liver and certain other tissues besides muscle. The expression level of MuRF2 in hepatic mononuclear cells (HMCs) is negatively associated with LPS/d‐GalN‐induced inflammatory liver injury. Further investigation suggests that MuRF2 inhibits the production of proinflammatory cytokines and cell migration by regulating NF‐κB.

## Materials and methods

### Mice and reagents

Male C57BL/6J mice, 6–8 weeks old, were obtained from the Animal Research Committee of the Institute of Biology and Cell Biology (Shanghai, China) and housed in a specific environment as previously described 21, 22. All animal experiments were undertaken in accordance with the National Institutes of Health *Guide for the Care and Use of Laboratory Animals*, with the approval of the Scientific Investigation Board of the Shandong Provincial Hospital Affiliated to Shandong University, Jinan, Shandong Province, China. LPS (*Escherichia coli*, 055:B5) and d‐GalN were obtained from Sigma‐Aldrich (St Louis, MO, USA; catalog no.: L2880 and G0500). Poly(I:C) was from InvivoGen (San Diego, CA, USA; catalog no.: tlrl‐picw). Mouse mAb to glyceraldehyde‐3‐phosphate dehydrogenase (GAPDH; catalog no.: 60004‐1‐lg), β‐actin (catalog no.: 60008‐1‐lg) and lamin B1 (catalog no.: 66095‐1‐lg) were purchased from Proteintech Group, Inc. (Rosemont, IL, USA). Anti‐MuRF2 (ab3) antibody produced in rabbit was purchased from Sigma‐Aldrich (catalog no.: SAB2102565). MuRF2 (C‐20) antibody was acquired from Santa Cruz Biotechnology (Dallas, TX, USA; catalog no.: sc‐49454); NF‐κB p65 (C22B4) rabbit mAb was obtained from Cell Signaling Technology (Danvers, MA, USA; catalog no.: 4764).

### Cell culture

The mouse macrophage cell line RAW264.7 were obtained from the American Type Culture Collection (Manassas, VA, USA) and cultured in Dulbecco's modified Eagle's medium (DMEM; Invitrogen, Carlsbad, CA, USA) containing 10% (vol/vol) fetal bovine serum (Gibco^®^ Sera, AUS; Invitrogen), 100 U·mL^−1^ penicillin and 100 μg·mL^−1^ streptomycin (Invitrogen). The cell lines were maintained at 37 °C in a humidified incubator with 5% CO_2_. RAW264.7 cells were stimulated with 100 ng·mL^−1^ LPS or 20 μg·mL^−1^ Poly(I:C) or were transfected with 2 μg Poly(I:C) by Lipofectamine‐2000 for different times.

### LPS/d‐GalN induced hepatitis and HMC collection

To induce hepatitis, 10 μg·kg^−1^ LPS and 800 mg·kg^−1^ d‐GalN in saline were i.p.‐injected into C57BL/6J mice. After 8 h, mice serum and liver tissue were collected and used in the subsequent experiments, including the evaluation of injury degree and the HMCs. HMCs were isolated by metrizamide gradient centrifugation as previously described 22.

### Hematoxylin and eosin staining

Liver tissues from different groups were fixed, embedded in paraffin and cut at 4 μm. The sections were taken off paraffin with xylene, hydrated with different concentration of ethanol and rinsed with distilled water. They were then hematoxylin stained for 5 min followed by 5 min rinsing with tap water. Similarly, they were eosin stained for 3 min followed by rinsing with tap water. A gradient (75, 85, 95 and 100%) concentration of alcohol was used to dehydrate. The slices were sealed by neutral gum with a coverglass and photographed under an optical microscope (Olympus, Tokyo, Japan).

### Alanine aminotransferase and aspartate aminotransferase detection

The serum levels of alanine aminotransferase (ALT) or aspartate aminotransferase (AST) were determined using the ALT or AST detection kit (Jiancheng Bioengineering Institute, Nanjing, China; catalog no.: C009‐2 or C0010‐2) according to the manufacturer's protocol.

### RNA extraction and quantification

Total RNA was extracted using RNAfast200 kit (Fastagen, Shanghai, China) according to the manufacturer's instructions, and reverse transcription was performed using a reverse transcription kit (TaKaRa, Kusatsu, Shiga, Japan). The expression of MuRF2, IL‐6 and interferon β (IFN‐β) was quantified using semiquantitative PCR or SYBR Premix Ex TapTM (TaKaRa) with β‐actin or GAPDH as an internal normalized reference. The specific sequences of primers used were as follows: 5′‐AGTTTGACACCCTCTACGC‐3′ (sense) and 5′‐TCTTGATGAGCTGCTTGG‐3′ (antisense) for MuRF1, 5′‐GACTGAAATGACCCAAGC‐3′ (sense) and 5′‐TTCCTGACTCCACCAACT‐3′ (antisense) for MuRF2, 5′‐CTAATCCTCTGTGGCAATCC‐3′ (sense) and 5′‐TCTCGTCCTCGTGCTCCT‐3′ (antisense) for MuRF3, 5′‐ACAACCACGGCCTTCCCTAC‐3′ (sense) and 5′‐CATTTCCACGATTTCCCAGA‐3′ (antisense) for IL‐6, 5′‐AGTTACACTGCCTTTGCC‐3′ (sense) and 5′‐GTTGAGGACATCTCCCAC‐3′ (antisense) for IFN‐β, 5′‐CAAGGTCATCCATGACAACTTTG‐3′ (sense) and 5′‐GTCCACCACCCTGTTGCTGTAG‐3′ (antisense) for GAPDH, 5′‐CCACACCCGCCACCAGTTCG‐3′ (sense) and 5′‐TACAGCCCGGGGAGCATCGT‐3′ (antisense) for β‐actin, 5′‐ATGAGCACTTCTCT GAATTACA‐3′ (sense) and 5′‐TTATTCATTTAGGGAATTCAACC‐3′ (antisense) for overall length of MuRF2 mRNA. Semiquantitative or quantitative PCR was performed as previously described 21, 23.

### Nuclear and cytoplasmic protein extraction

NE‐PER Nuclear and Cytoplasmic Extraction Reagents (Thermo Fisher Scientific, Waltham, MA, USA; catalog no.: 78835) were used according to the manufacturer's instructions.

### Western blot assay

The cultured cells were washed with cold PBS and lysed with RIPA lysis buffer (Beyotime Biotechnology, Shanghai, China) supplemented with a protease inhibitor ‘cocktail’. After the protein concentrations were measured using the bicinchoninic acid assay (Thermo Fisher Scientific), equal amounts of protein lysates were separated by SDS/PAGE and transferred to poly(vinylidene difluoride) membranes (Millipore, Billerica, MA, USA) for immunoblot analysis as described previously 24. The membranes were then incubated with the primary antibodies overnight at 4 °C. After washing with tris buffered saline with tween, the membranes were hybridized with the corresponding horseradish peroxidase‐conjugated secondary antibody (Santa Cruz Biotechnology). Finally, an enhanced chemiluminescence reagent kit (Millipore) was used to detect the objective protein in accordance with the manufacturer's protocol.

### Immunofluorescence analysis

RAW264.7 cells were grown on coverslips overnight and stimulated with 100 ng·mL^−1^ LPS or 20 μg·mL^−1^ Poly(I:C) for 8 h. The cells were fixed in 4% paraformaldehyde for 15 min and permeabilized with 0.1% Trition X‐100. After blocking with 10% BSA in PBS for 2 h at room temperature, they were incubated with primary antibodies against MuRF2 for 1.5 h at room temperature and washed three times with PBS for 5 min per time. Cells were incubated with secondary antibodies (catalog no.: CA11012s; Invitrogen) for 1 h at room temperature in the dark and washed 3 times with PBS for 5 min per time. Cell nuclei were stained with 4′,6‐diamidino‐2‐phenylindole (DAPI) for 8 min and wash three times with PBS for 5 min per time. Neutral gum was used to seal the coverslips and the images were captured using a high sensitivity laser scanning confocal microscope (LSM780; Zeiss, Oberkochen, Germany) with the appropriate filters and laser (561 and 633 nm) and a ×63 objective lens.

### Assessment of macrophage migration

A Boyden chamber was used to observe macrophage migration as we have previously described 21. Briefly, RAW264.7 cells before or after MuRF2 overexpression were resuspended in 200 μL of DMEM with or without LPS, and then seeded into the upper chamber. After incubation for 20 h at 37 °C in a humidified incubator with 5% CO_2_, the Boyden chamber was fixed and stained. Macrophage migration was observed and photographed with an optical microscope.

### Statistical analysis

All data were presented as results from three or four independent experiments. All data were expressed as the mean ± SD. Numeric comparison between two groups were analyzed by one‐way ANOVA and two‐tailed Student's *t* test. Correlation between MuRF2 expression and ALT or AST level was analyzed with Spearman's rank test. In all cases, a value of *P* < 0.05 was considered statistically significant.

## Results

### MuRF2 is widely expressed in various tissues, besides skeletal muscle and heart

**Figure 1:**
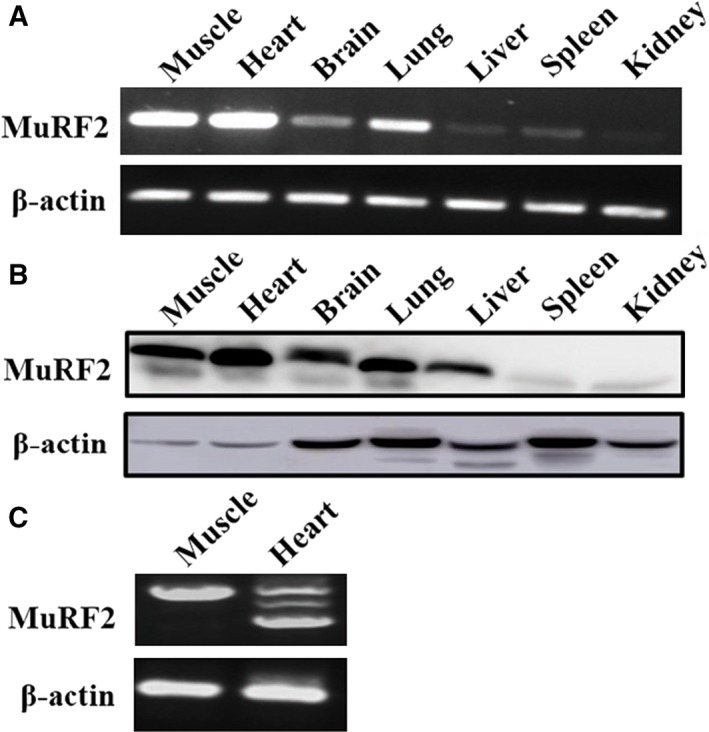
MuRF2 is expressed in mouse in multiple tissues, besides muscle and heart. (A,B) MuRF2 expression was detected with semiquantitative PCR (A) and western blot analysis (B). (C) Overall‐length primers of MuRF2 mRNA were used to observe its isoforms in the mouse skeletal muscle and heart by semiquantitative PCR.

Muscle RING‐fingers have been thought of as proteins expressed specifically in striated muscle, where they play an important role in pathology and physiology, including muscle protein turnover and atrophy 9, 25 and myocardial hypertrophy and infarct 26, 27. However, less is known regarding the expression and potential roles of MuRF2 in other tissues. Therefore, we first detected the expression of MuRF2 in certain key tissues and organs in mice, such as brain, lung, liver, spleen and kidney, at the protein and mRNA level, with muscle and heart serving as positive controls. Results showed that MuRF2 was indeed expressed in other tissues besides muscle and heart (Fig. 1A,B). When the full‐length primer was used in RT‐PCR, three spliceosomes of MuRF2 were shown in the mouse heart and one in the skeletal muscle (Fig. 1C). This result was inconsistent with the NCBI‐GenBank database, where there is only one reported transcript (NM_001081281.1) in mice. Taken together, our results suggest that MuRF2 is universally expressed in other tissues besides striated muscle.

### MuRF2 expression decreases in HMCs of mice with LPS/d‐GalN‐induced hepatitis

**Figure 2:**
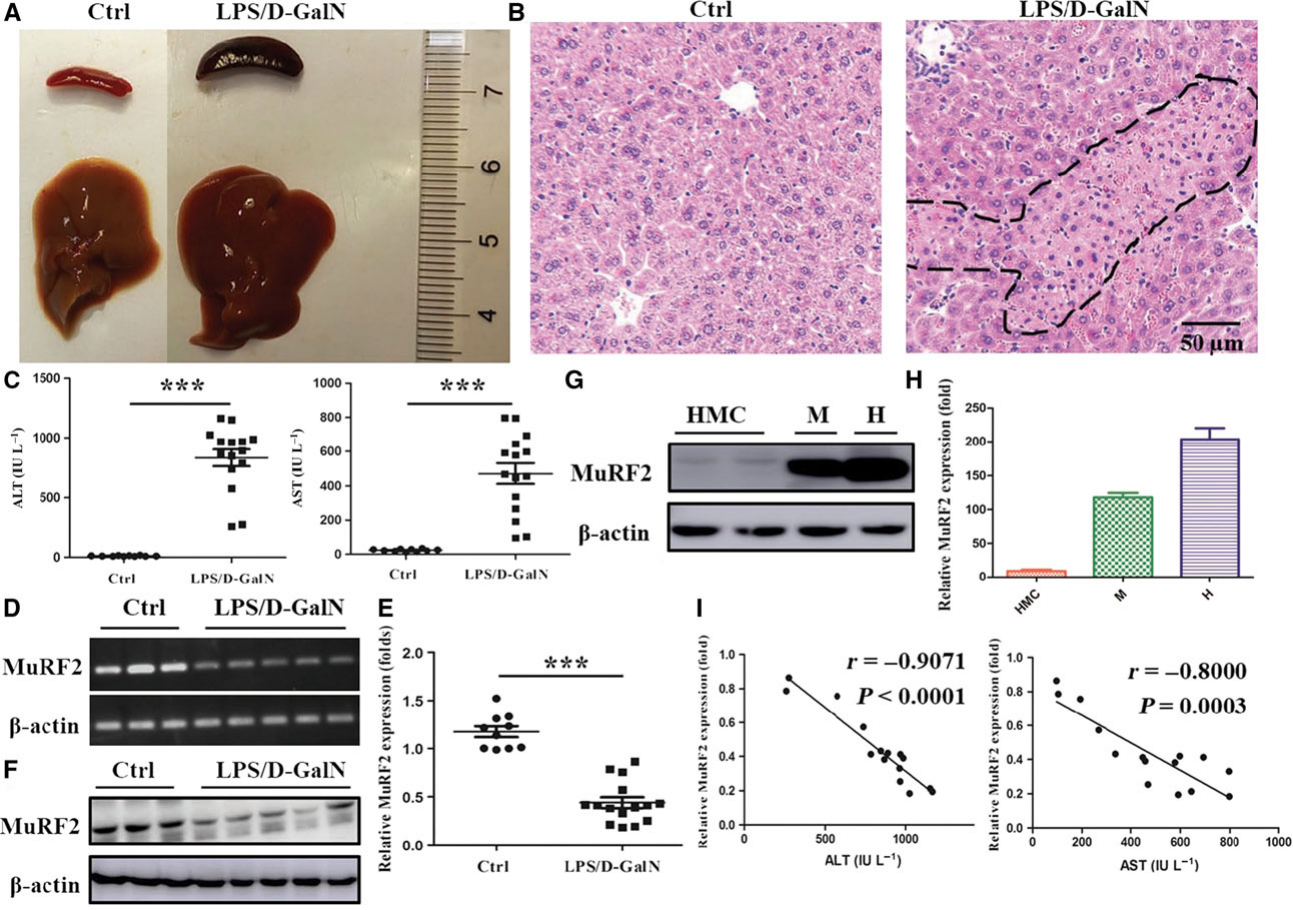
MuRF2 expression decreases in HMCs of LPS/d‐GalN‐treated mice and is inversely correlated with serum ALT and AST content. Mice were killed after being administered with i.p. LPS and d‐GalN for 8 h. (A) Gross images of liver and spleen. (B) The liver tissue was embedded, sliced and stained with hematoxylin and eosin and photographs were obtained with an optical microscope (the dashed line shows liver tissues necrosis area; ×100 original magnifications). (C) The serum levels of ALT or AST were determined. (D,E) Semiquantitative (D) or quantitative (E) PCR was performed to determine MuRF2 expression in HMCs of LPS/d‐GalN‐treated mice. β‐Actin was an internal normalized reference. (F,G) Western blot was used to detect MuRF2 expression in HMCs, muscle (M) and heart (H) in mice. β‐Actin was an internal normalized reference. (H) Quantitative PCR were performed to determine MuRF2 expression in HMCs, muscle (M) and heart (H) of mice. (I) The correlation was determined between MuRF2 mRNA expression in HMCs and serum ALT or AST levels. Each data point represents one mouse. ***P < 0.001.

Previous studies from our group and others have demonstrated that macrophages play an essential role in hepatitis 22. Moreover, Lopez *et al*. 28 validated that in healthy rodent livers, there are 20–40 macrophages for every 100 hepatocytes. Hence, in order to investigate the role of MuRF2 in hepatitis, we established a mouse hepatitis model with LPS/d‐GalN as previously reported 11, 12. Next, we evaluated the expression of MuRF2 in HMCs and analyzed the correlation of MuRF2 expression with ALT or AST. We observed that the liver and spleen from mice with hepatitis were larger, more purple and dimmer than those of health mice (Fig. 2A). In addition, we determined liver histological changes with hematoxylin and eosin staining and observed that there was obvious hyperemia, swelling, inflammatory cell infiltration and necrosis in hepatic livers compared with those of health mice (Fig. 2B). Consistent with these histological results, the serum levels of ALT and AST in mice with hepatitis were significantly increased compared with that of the control (*P* < 0.001, Fig. 2C). These data showed that we successfully established an LPS/d‐GalN‐induced hepatitis mouse model. Subsequently, we collected the HMCs and detected expression levels of MuRF2 at the mRNA and protein level. Compared with the control group, MuRF2 expression levels were significantly decreased in the induced hepatitis model (*P* < 0.001, Fig. 2D–F). In addition, we observed MuRF2 expression in HMCs compared with muscle and heart. As shown in Fig. 2F,H, MuRF2 expression in HMCs was lower than in muscle and heart in mice. Further analysis indicated that the expression level of MuRF2 in HMCs was inversely associated with the serum levels of ALT and AST in mice with hepatitis with Spearman's *r* of −0.9071 and −0.8000, respectively (Fig. 2I). Taken together, these results suggest that the decrease of MuRF2 expression in the HMCs of LPS/d‐GalN‐induced hepatitis was negatively correlated with liver injury indexes, including ALT and AST.

### Activation of the LPS/TLR4 pathway inhibits MuRF2 expression and promotes its nuclear translocation in RAW264.7 cells

**Figure 3:**
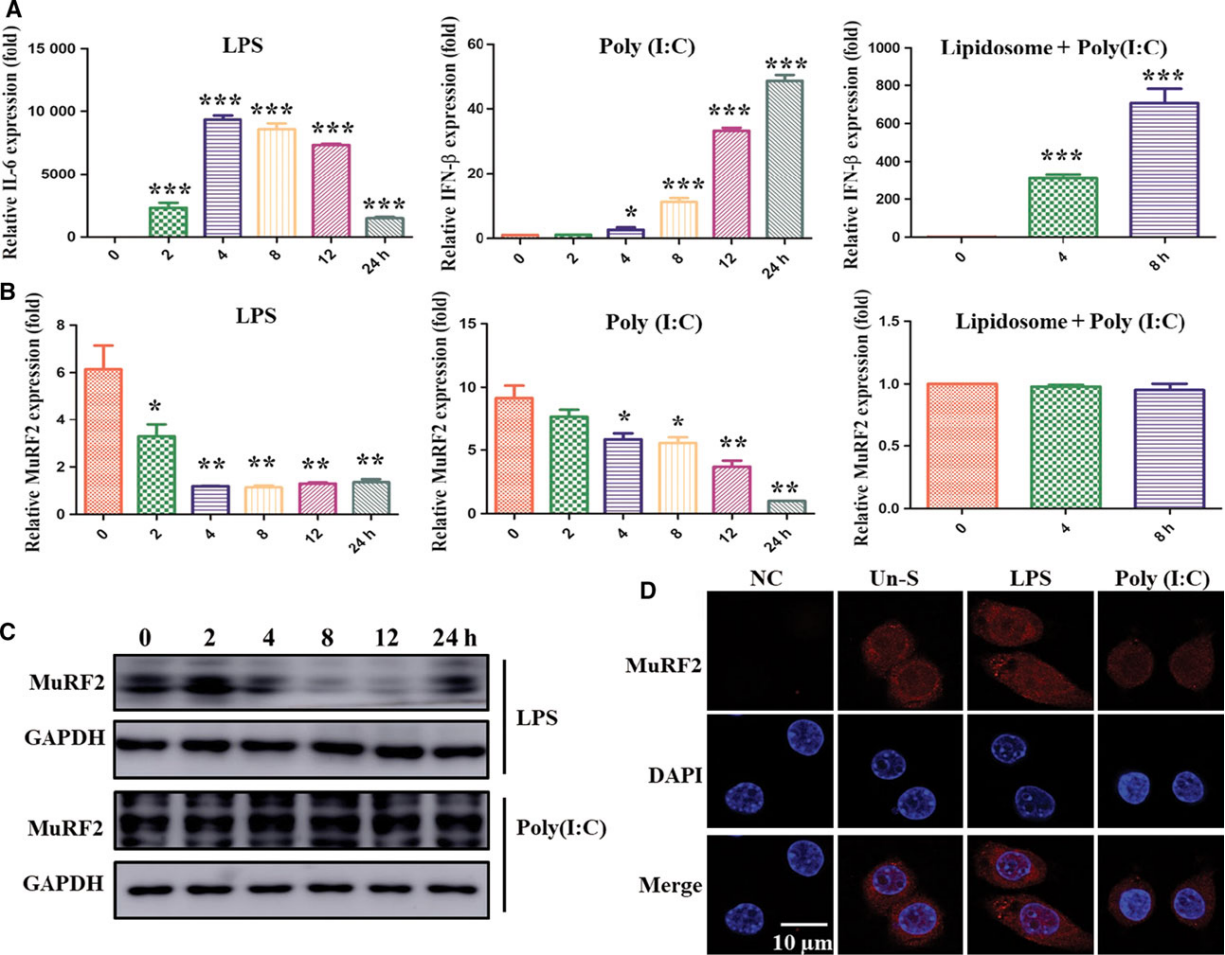
LPS stimulation leads to down‐regulation of MuRF2 expression in RAW264.7 macrophages, but not Poly(I:C) stimulation or transfection. (A) RAW264.7 cells were treated at different time points with LPS (100 ng·mL ^−1^), Poly(I:C) (20 μg·mL ^−1^) or lipidosome plus Poly(I:C) (2 μg). IL‐6 and IFN‐β expression was observed with quantitative PCR. (B,C) MuRF2 expression was determined by quantitative PCR (B) and western blot (C). GAPDH was an internal normalized reference. (D) After RAW264.7 cells were stimulated for 8 h with LPS [100 ng·mL ^−1^, pretreated for 30 min with proteasome inhibitor MG132 (10 μm)] or Poly(I:C) (20 μg·mL ^−1^), endogenous MuRF2 was located using a confocal microscope (NC, negative control; Un‐S: unstimulated). DAPI, 4′,6‐diamidino‐2‐phenylindole. *P < 0.05, **P < 0.01, ***P < 0.001.

To explore the effect of MuRF2 on macrophages, we treated RAW264.7 cells with common activators, such as LPS (TLR4), Poly(I:C) (TLR3) and lipidosome plus Poly(I:C) (retinoic acid‐inducible gene I; RIG‐I). As shown in Fig. 3A, the expression of IL‐6 and IFN‐β was significantly increased in TLR3/4 and RIG‐I groups, respectively. In addition, these three agonists can effectively activate RAW264.7 macrophages. We measured MuRF2 mRNA expression levels when RAW264.7 was stimulated with these three agonists. As shown in Fig. 3B, the expression of MuRF2 was reduced in the LPS and Poly(I:C) treatment groups. However, expression did not change in the lipidosome plus Poly(I:C) group. At the same time, the expression of MuRF1 and ‐3 was not detectable at all by qPCR in the LPS and Poly(I:C) groups (data not shown). Further detection of protein levels by western blot analysis showed that the expression of MuRF2 was markedly decreased only in LPS‐induced macrophage activation, rather than Poly(I:C) (Fig. 3C). Thus, to examine the location of MuRF2 expression by immunofluorescence assay, we pretreated RAW264.7 macrophages with *N*‐carbobenzoxyl‐l‐leucinyl‐l‐norleucinal (MG132), a proteasome inhibitor, for 30 min and then stimulated with LPS for 8 h. As shown in Fig. 3D, MuRF2 was expressed in the cytoplasm and the nucleus in the absence of stimulation. However, when RAW264.7 cells were stimulated with LPS, not Poly(I:C), MuRF2 would translocate into the nucleus. Taken together, these data show that the expression level of MuRF2 is down‐regulated by LPS‐induced macrophage activation. LPS‐induced macrophage activation also causes MuRF2 to localize to the nucleus.

### MuRF2 overexpression inhibits the production of inflammatory cytokines and cell migration in LPS‐induced RAW264.7

**Figure 4:**
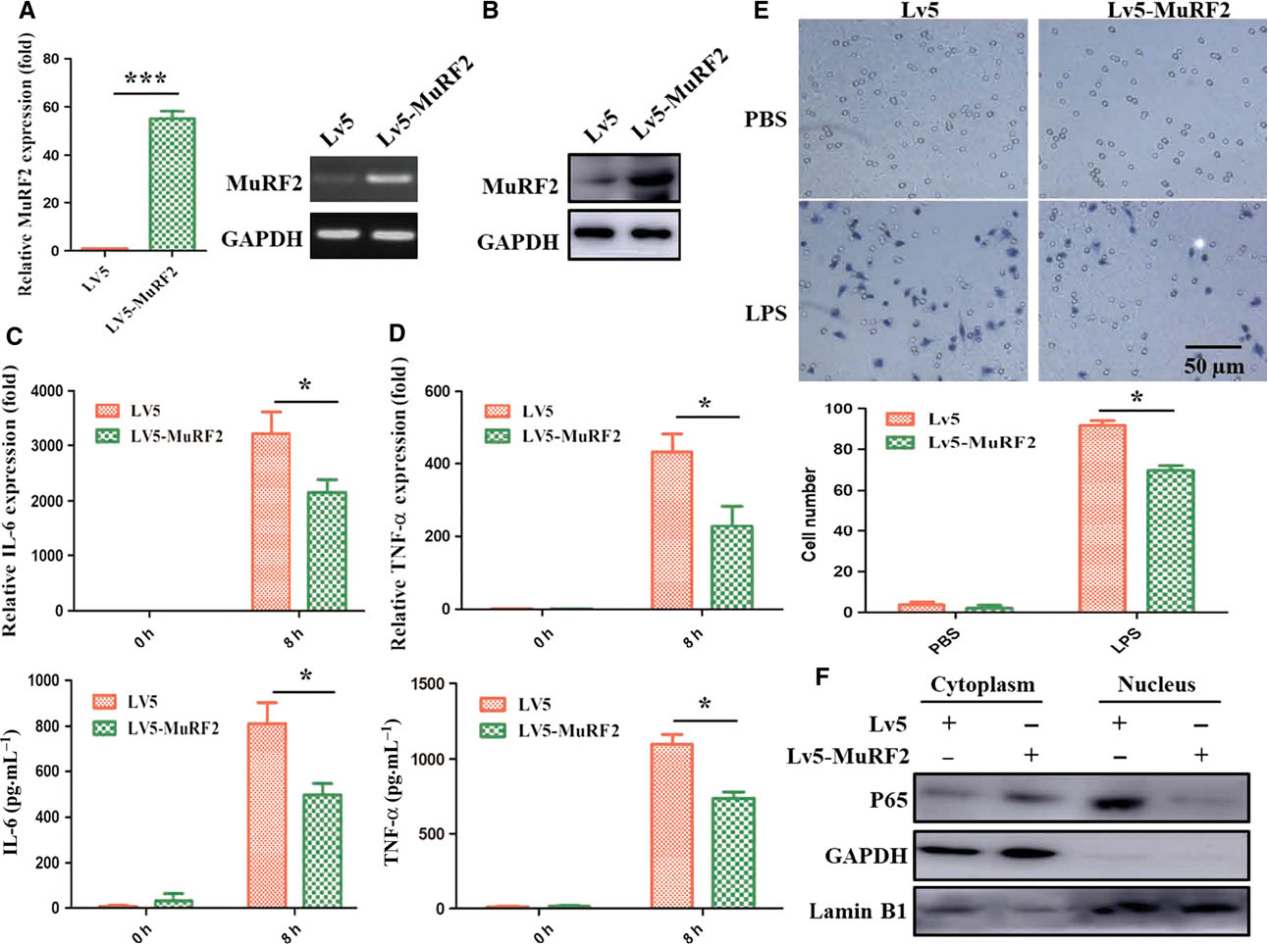
MuRF2 overexpression inhibits the production of inflammatory cytokines and cell migration in LPS‐induced RAW264.7. Control (LV5) or MuRF2 (LV5‐MuRF2) transfected RAW264.7 cells were screened with puromycin to acquire stable expression macrophages. (A,B) MuRF2 expression was estimated by PCR (A) and western blot (B). (C,D) The expression and secretion of IL‐6 and TNF‐α were monitored by PCR and ELISA. (E) After the stable expression, RAW264.7 cells were treated for 20 h with 100 ng·mL ^−1^ LPS. Migrated cells on the lower surface of the Boyden chamber were stained with hematoxylin (upper panel, ×200 original magnification). Additionally, the average number of the cells per field was counted: ×10 objective magnifications from five microscopy fields in three independent experiments (lower panel). (F) Nuclear and cytoplasmic proteins were extracted. P65 expression was analyzed by western blot. GAPDH and lamin B1 were internal normalized references. *P < 0.05, ***P < 0.001.

When a pathogen invades an organism, macrophages are recruited by migration and release cytokines to induce an inflammatory response. To identify the roles of MuRF2 in macrophages, we observed the production of IL‐6 and cell migration by gain of function. As shown in Fig. 4A,B, MuRF2 expression was successfully elevated at the mRNA and protein level in RAW264.7 cells by Lv5‐MuRF2 transfection. These cells were subsequently stimulated with LPS for 8 h, and the expression and secretion of IL‐6 and TNF‐α were measured. As shown in Fig. 4C,D, the production of IL‐6 and TNF‐α was significantly decreased compared with the control (*P* < 0.05). Similarly, the migration rate of LPS‐activated RAW264.7 cells was also obviously lower in the MuRF2 overexpression group than the control (Fig. 4E).

Nuclear factor‐κB, composed of p65 and p50, is a common downstream nuclear transcription factor in the LPS‐induced macrophage activation pathway 29. To explore the mechanism of MuRF2 in macrophage activation, the expression of p65 in both the cytoplasm and the nuclei of RAW264.7 cells were evaluated after MuRF2 overexpression. The results showed that p65 expression level in the nucleus of the MuRF2 overexpression group was remarkably decreased compared with that of the control group, while no obvious change occurred in the cytoplasm (Fig. 4F). Taken together, these results suggest that MuRF2 might prevent macrophages from producing inflammatory factors and migrating by down‐regulating the level of nuclear NF‐κB.

## Discussion

The MuRF family has been extensively studied in striated muscle, and has been demonstrated to play important roles in several biological processes of muscle including signal transduction to maintain muscle structure and function by mediating ubiquitination of target proteins 25. Furthermore, MuRF2, as one member of this family, has been shown to regulate the nuclear transcription factors SRF and PPAR‐γ1 in cardiac myocytes 10, 30, which made us question whether MuRF2 may play a part in other tissues and cells by regulating other nuclear transcription factors.

We were the first to find that MuRF2 is also expressed in tissues other than muscle, including liver. Our further investigation of its roles in LPS/d‐GalN‐induced hepatitis revealed a significantly negative correlation between MuRF2 expression level of HMCs and the serum levels of ALT and AST in mice with hepatitis. This correlation indicates that MuRF2 may attenuate LPS/d‐GalN‐induced hepatitis through affecting the functions of macrophages.

Lipopolysaccharide/d‐GalN‐induced acute hepatitis simulates the pathological process of clinical acute hepatitis resulting from sepsis and endotoxemia 31. In this model, the degree of liver injury largely depends on the activity of macrophages. Activated by LPS, macrophages produce chemokines and proinflammatory cytokines, which mediate the migration of macrophages and cause a cascade of events leading to inflammatory injury 32. Our study showed the production of IL‐6 and migration rate of LPS‐activated RAW264.7 cells are significantly reduced after MuRF2 overexpression. This finding means that MuRF2 can inhibit macrophages activated by LPS. This phenomenon was specific because no significant changes in MuRF2 expression level were demonstrated in RAW264.7 cells when stimulated with Poly(I:C) or lipidosome plus Poly(I:C).

Muscle RING‐finger 2 is primarily found in the cytoplasm, although previous work demonstrated that MuRF2 also plays a role in the nucleus. Lange *et al*. 10 found that MuRF2 could translocate into nuclei and regulate the activity of the nuclear transcription factor SRF in myocytes. Coincidentally, another study performed by He *et al*. 30 identified that MuRF2 could also regulate the nuclear transcription factor PPAR‐γ1 in cardiomyocytes. Our study found that MuRF2 translocated into nuclei in LPS‐stimulated RAW264.7 cells compared with control groups. However, whether MuRF2 might play a part in the downstream signal transduction in macrophages and cardiomyocytes should be further confirmed.

Nuclear factor‐κB, a heterodimer composed of p65 and p50, is a key regulator of inflammation. Combining with the inhibitor of NF‐κB (IκB) as an inactive form in the cytoplasm, NF‐κB is activated by the IκB kinase complex with free NF‐κB released, which then translocates into the nucleus and binds to specific DNA sites to regulate transcription 33. Traditional inflammatory inhibitors, such as salicylates, non‐steroidal anti‐inflammatory drugs and glucocorticoids, are mostly based on the regulation of NF‐κB activation. Recently, termination of NF‐κB activity has drawn increasing attention. In addition to the traditional export back to cytoplasm, the ubiquitination mediated by E3 ubiquitin ligases and the subsequent proteasomal degradation of p65 has been investigated as another vital termination mechanism. Tanaka *et al*. 18 reported that PDLIM2, as a nuclear E3 ubiquitin ligase, can target the p65 subunit and mediate its polyubiquitination, thereby terminating the activity of NF‐κB through subsequent degradation. Another study conducted by Ryo *et al*. 19 indicated that SOCS‐1 could modulate the ubiquitination and subsequent degradation of p65, acting as an E3 ubiquitin ligase. In addition, Hou *et al*. 20 found that another E3 ubiquitin ligase, PPAR‐γ, could induce the ubiquitination and degradation of nuclear p65 as well as reduce cytoplasmic p65 translocating to the nucleus. In the present study, we found that the expression level of p65 in nuclei of RAW264.7 cells was decreased after MuRF2 overexpression, which may be evidence for the assumption that MuRF2 terminates NF‐κB activity through a ubiquitin‐dependent proteasome degradation pathway.

In summary, we first demonstrated that MuRF2 is expressed not only in the muscle but also in other tissues, including liver. In addition, our results indicate that the expression of MuRF2 is significantly decreased in HMCs of mice with LPS/d‐GalN‐induced hepatitis and negatively correlated with the serum ALT and AST in mice with hepatitis. Moreover, MuRF2 is translocated from the cytoplasm into the nucleus in LPS‐treated RAW264.7 cells; the cytokine production and macrophage migration are decreased by MuRF2 gain of function; and p65 level in nuclei was greatly reduced when MuRF2 was overexpressed. These findings suggest that MuRF2 may inhibit the functions of macrophages by terminating NF‐κB activity to alleviate immune‐mediated hepatitis.

## Author contributions

Conceived and designed the experiments: JQ, HB and CQ. Performed the experiments: HB, DZ and SG. Analyzed the data: HB and FL and TL. Contributed reagents/materials/analysis tools: QZ, XL, SS and SS. Wrote the paper: QZ, WR and JQ.
